# Supplementary material for: Neuronal parts list and wiring diagram for a visual system
Source: Nature. 2024 Oct 2;634(8032):166–80. doi: 10.1038/s41586-024-07981-1 (PMC11446827; doi:10.1038/s41586-024-07981-1)
Supplement: Supplementary file 5 — Cards for all cell types. Each card has 6 panels (clockwise): logical connectivity predicate diagram, numeric stats, illustration of a typical cell, followed by 3D rendering of all cells from three orthogonal viewpoints. Also available at Codex. [file 41586_2024_7981_MOESM5_ESM.zip › pdf/C2.pdf]

C2

predicate precision: 97%, recall: 88%

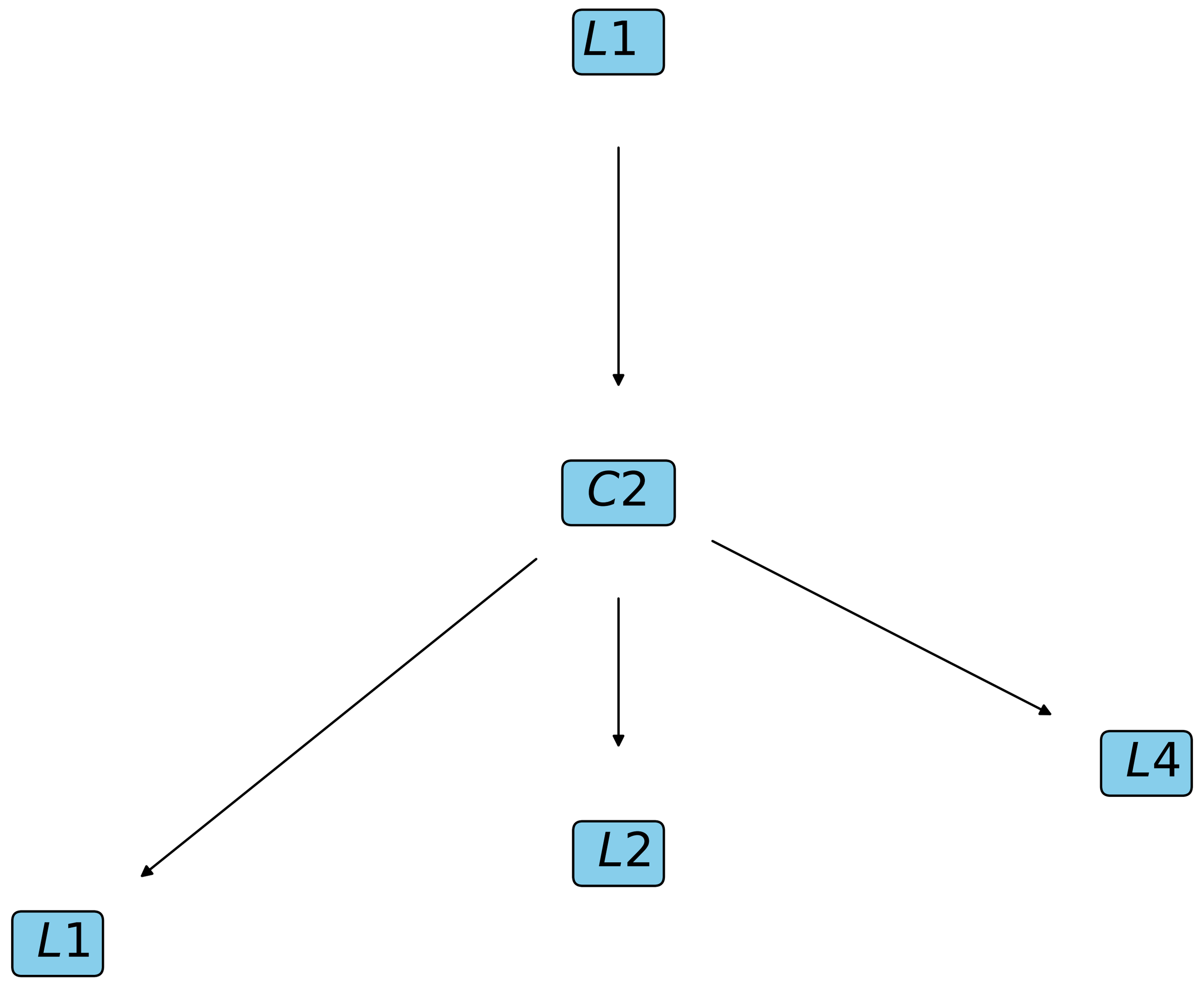

avg. stats

cells	743
NT prediction	GABA (59%)
top in/out regions	ME (92%) / ME (80%)
in/out degree	17 / 12
in/out synapses	94 / 103
cable len	469μ
surface	858μ^2
volume	64μ^3

morphology

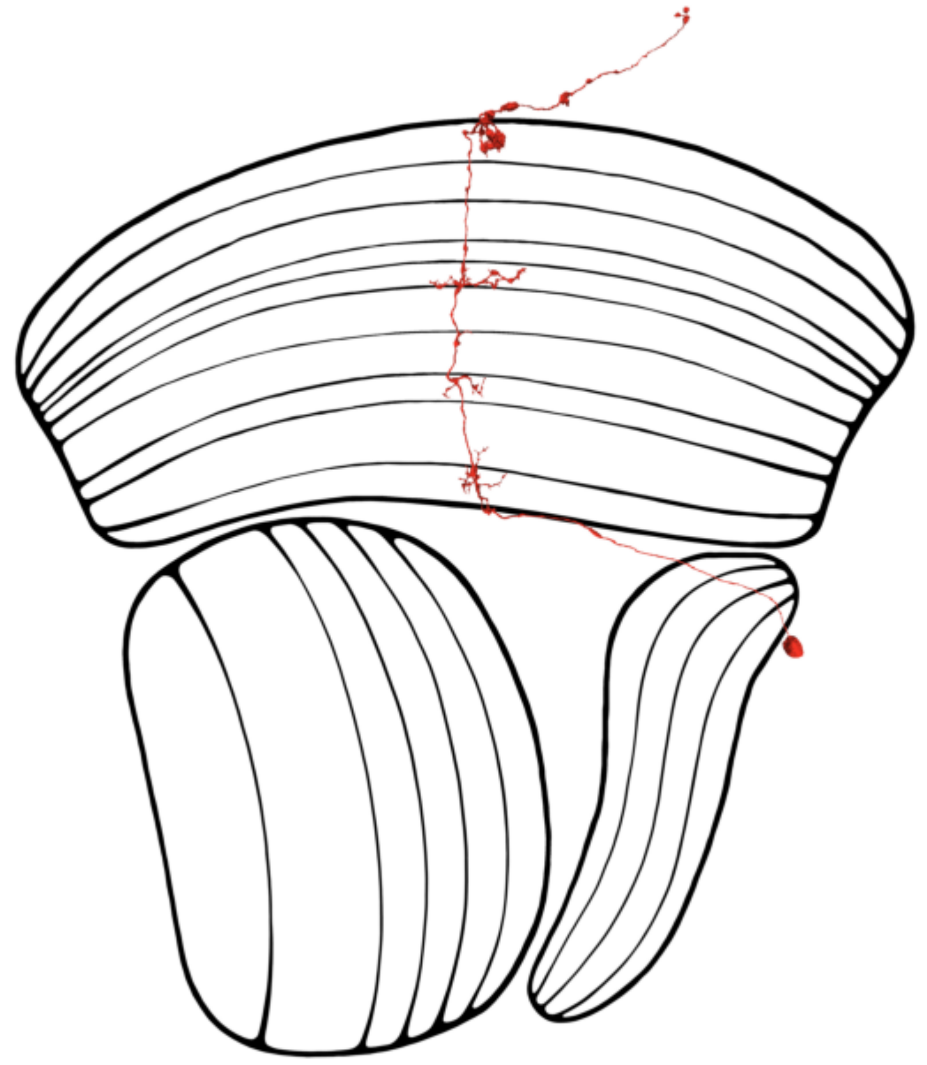

anterior view

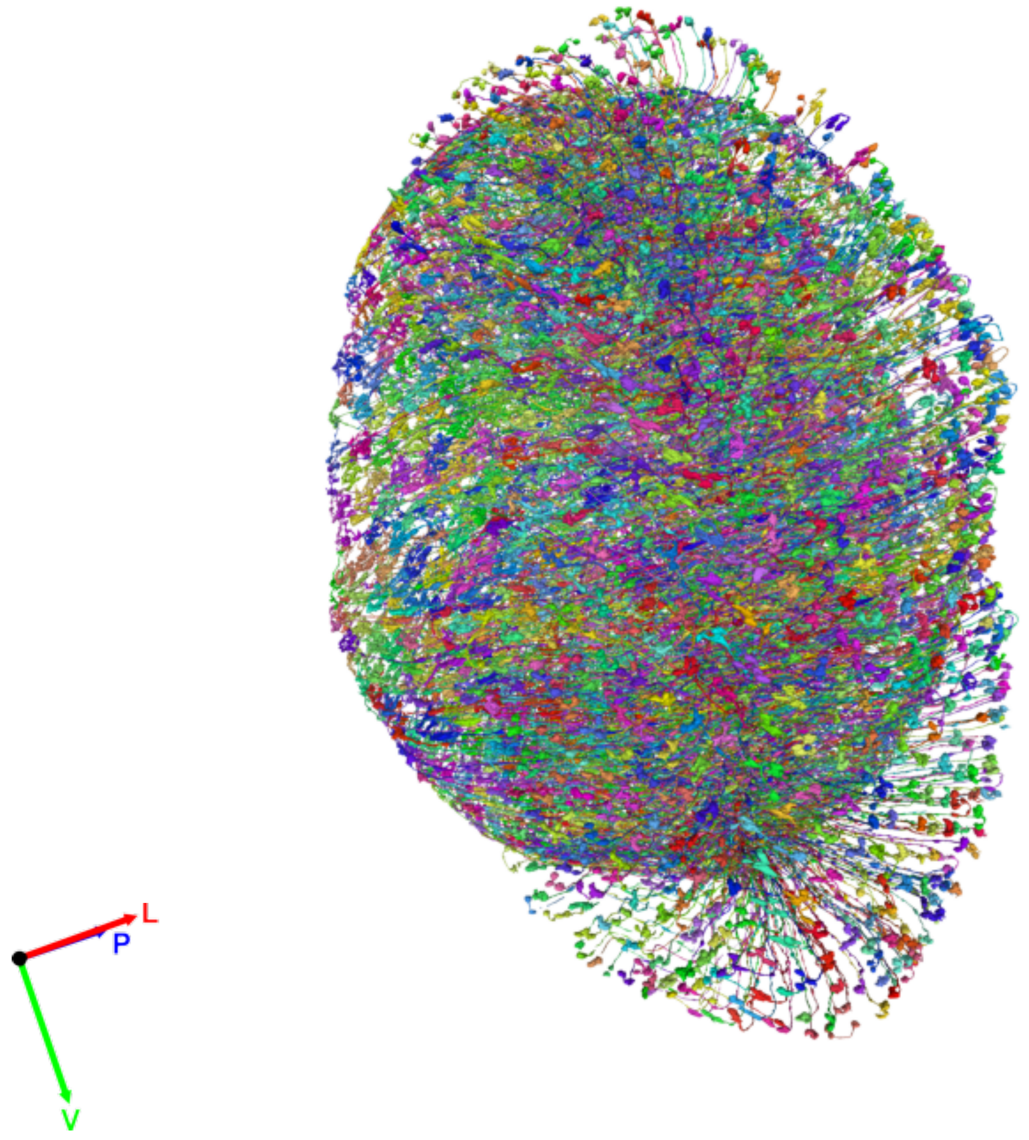

ventral view

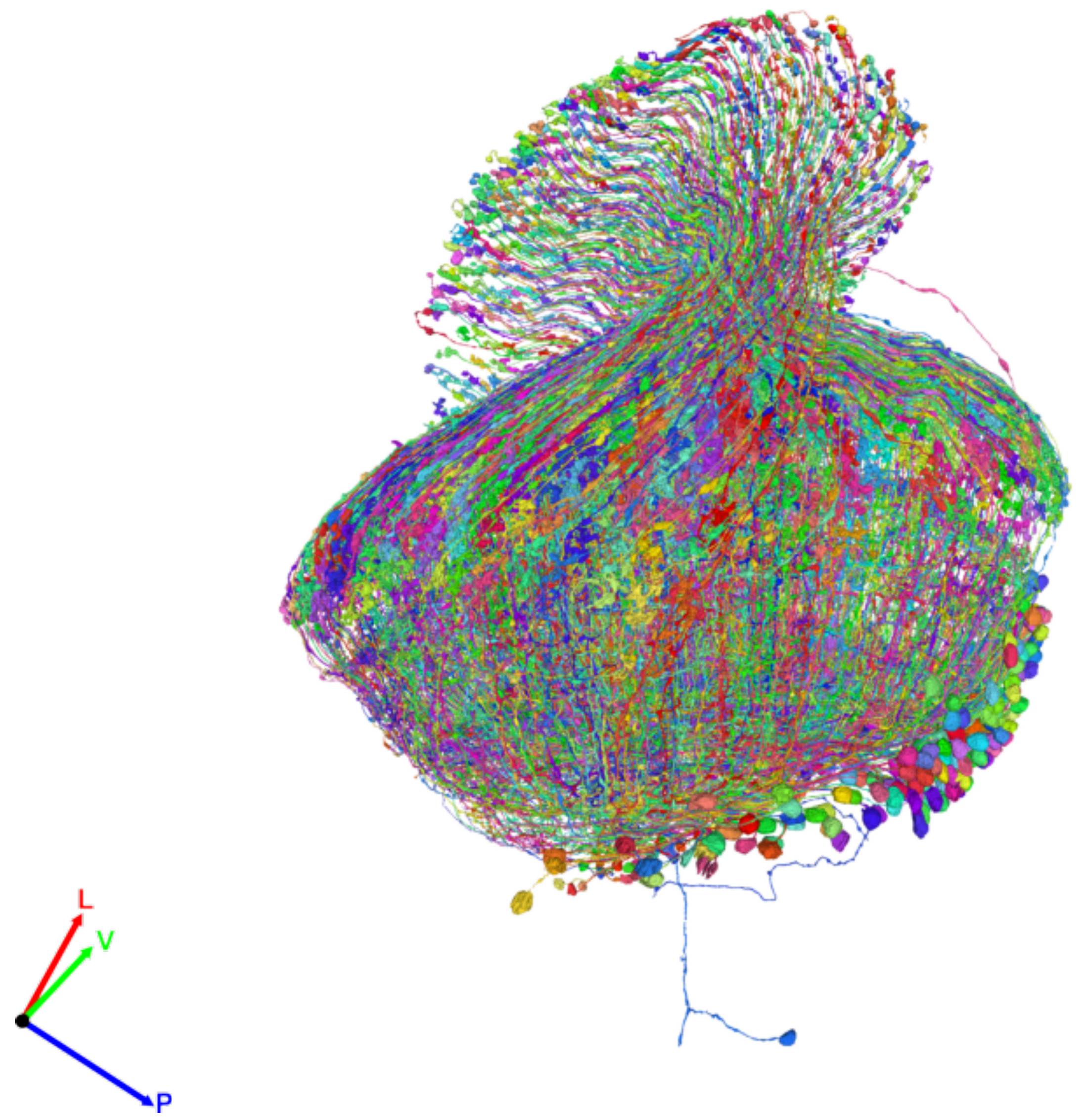

side view

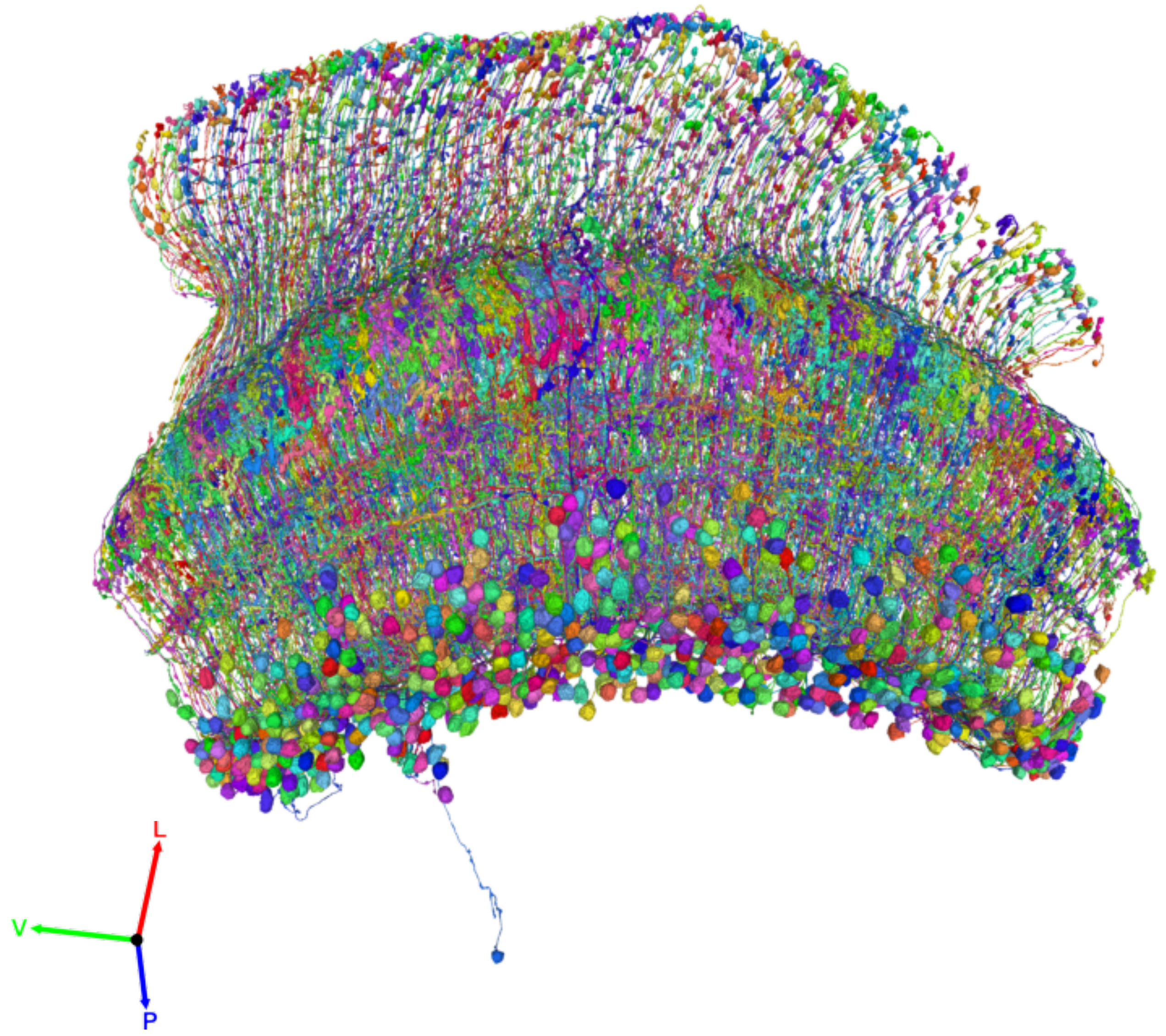

Centrifugal
